# pH Responsive Hydrogels for the Delivery of Capecitabine: Development, Optimization and Pharmacokinetic Studies

**DOI:** 10.3390/gels8120775

**Published:** 2022-11-28

**Authors:** Umaira Rehman, Rai Muhammad Sarfraz, Asif Mahmood, Shehla Akbar, Ahmed E. Altyar, Roaa M. Khinkar, Heba A. Gad

**Affiliations:** 1 Department of Pharmaceutics, Faculty of Pharmacy, University of Sargodha, Sargodha 40100, Pakistan; 2 Department of Pharmacy, The University of Chakwal, Chakwal 48800, Pakistan; 3 Allama Iqbal Campus, Punjab University College of Pharmacy, University of the Punjab, Lahore 54000, Pakistan; 4 Department of Pharmacognosy, Faculty of Pharmacy, The Islamia University of Bahawalpur, Bahawalpur 63100, Pakistan; 5 Department of Pharmacy Practice, Faculty of Pharmacy, King Abdulaziz University, P.O. Box 80260, Jeddah 21589, Saudi Arabia; 6 Pharmacy Program, Batterjee Medical College, P.O. Box 6231, Jeddah 21442, Saudi Arabia; 7 Department of Pharmaceutics and Industrial Pharmacy, Faculty of Pharmacy, Ain Shams University, Cairo 11566, Egypt

**Keywords:** capecitabine, sustained release, hydrogel, pharmacokinetic, drug discovery, health care

## Abstract

The objective of the current study was to achieve a sustained release profile of capecitabine (CAP), an anticancer agent frequently administered in conventional dosage form due to its short plasma half-life. A drug-loaded smart pH responsive chitosan/fenugreek-g-poly (MAA) hydrogel was synthesized by an aqueous free radical polymerization technique. The developed network was evaluated for capecitabine loading %, swelling response, morphology, structural and compositional characteristics, and drug release behavior. Significantly higher swelling and in vitro drug release rate were exhibited by formulations at pH 7.4 than at pH 1.2, demonstrating the pH responsive character of hydrogels. Swelling percentage and CAP loading ranged within 74.45–83.54% and 50.13–72.43%, respectively. Maximum release, up to 93%, was demonstrated over 30 h, evidencing the controlled release pattern of CAP from hydrogels. The optimized formulation was further screened for acute oral toxicity studies. No signs of oral, dermal, or ocular toxicities were noticed, confirming safety evidence of the network. Furthermore, pharmacokinetic analysis demonstrated the sustained release response of CAP from hydrogels as confirmed by a significant increase in plasma half-life (t_1/2_) (13 h) and AUC (42.88 µg h/mL) of CAP. Based on these findings, fabricated hydrogels are strongly recommended as a biocompatible carrier for colorectal delivery of active agents.

## 1. Introduction

Capecitabine (CAP) is a potent anticancer agent often recommended for treating colorectal cancer at 1250 mg/m^2^ twice daily. It exerts its action by being converted into its active metabolite, i.e., 5-fluorouracil, by enzymatic action in the tumor cells. Here, it inhibits DNA synthesis, slowing down the tumor growth. Despite having strong anticancer potential, it exhibits certain limitations owing to short plasma half-life, fluctuations in plasma concentration, and rapid clearance from the body. Furthermore, adverse effects are associated with its therapeutic regimen (1250 mg/m^2^ two times a day), resulting in patient non-compliance behavior. These limitations necessitate the development of a sustained release delivery system that would improve its therapeutic effect and patient compliance [1].

Recently, various drug delivery techniques have been evaluated for the controlled release potential of drugs, particularly chemotherapeutic agents. One of the drug delivery strategies is hydrogel, having fabulous characteristics. Hydrogels are polymer-based cross-linked networks comprising excess water or physiological fluid capable of releasing the drug slowly [2,3]. They have good drug encapsulating efficiency, excellent biocompatibility, and non-cytotoxicity, rendering them favorable for different applications, including drug delivery, stem cell research, tissue engineering, and cell culture [4,5]. Currently, various forms of hydrogels are being developed, including microparticles [6], nanoparticles [7], matrices, composites [8], and discs and films, depending upon desired utilization.

Chitosan is a cationic polysaccharide that contains D-glucosamine and N-acetyl-D-glucosamine units having β-(1-4) linkages in its structure. Solubility of chitosan in dilute acidic solutions is assigned to the protonation of its primary amine groups. Owing to its biocompatibility and non-toxicity, chitosan is widely used as a polymer for the fabrication of hydrogels [9].

Fenugreek seed gum is obtained by extracting the endosperm of fenugreek (*Trigonella foenum-graecum* L.), where the main constituents of the gum are polysaccharides. The high content of gum present in fenugreek seeds converts into a thick and viscous mass on exposure to water and biological fluids. Fenugreek seed gum has numerous applications in the food industry and pharmaceutical formulations as a binding, suspending, and gelling agent [10]. Here, the gum is used as polymer, along with chitosan, to augment the viscosity of the formed gel and to explore swelling and release potentials.

Methacrylic acid (MAA) is widely employed as a pH responsive monomer in the synthesis of hydrogels. At acidic pH, carboxylic groups within the monomer induce shrinkage of the network, whereas in basic media ionization, they cause repulsion within the groups, and appreciable swelling response results [11].

In our present work, CAP-loaded pH responsive chitosan/fenugreek-g-poly (MAA) hydrogels have been synthesized through the free radical polymerization technique using polymers, monomers, and cross-linking agents in varying proportions. The study aimed to attain the controlled delivery of CAP via the oral route to minimize its oral limitations and improve patient compliance. The safety of the grafted network was investigated. In addition, in vivo pharmacokinetic studies were carried out in healthy male rabbits to investigate the sustained release response of CAP-loaded hydrogels.

## 2. Results and Discussion

### 2.1. CAP Loading

**Figure 1:**
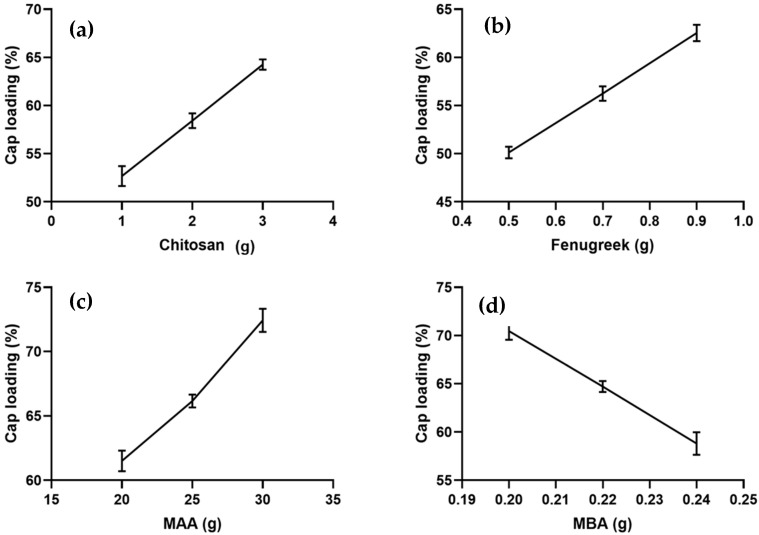
Effect of ingredients concentration on CAP loading (**a**) Chitosan, (**b**) Fenugreek, (**c**) Methacrylic acid (MAA) and (**d**) N,N-methylene bisacrylamide (MBA).

Chitosan/fenugreek-g-poly (MAA) hydrogels were loaded with CAP using a swelling diffusion approach. Drug loading percentage was significantly affected by the sequentially rising hydrogels’ chitosan, fenugreek, MAA, and MBA ratios. CAP loading percentage ranged from 50.13–72.43%, as shown in Figure 1. CAP loading was observed to be directly related with the swelling capacity of hydrogels.

Formulations CHF1-CHF3, CHF4-CHF6, and CHF7-CHF9, containing increasing concentrations of chitosan, fenugreek, and MAA, displayed increasing drug loading, i.e., 52.67–64.25%, 50.13–62.55%, and 61.51–72.43%, respectively, as shown in Figure 1a–c. Ionized state of functional units from both polymers (amino group from chitosan and hydroxyl group from fenugreek) and monomer (carboxylic) at pH 7.4 created electrostatic repulsion within the groups, thus allowing the intake of more fluid inside the network [12]. So, higher loading and swelling was observed upon increasing the ratios of both polymers and monomer.

Formulations CHF10-CHF12, having increasing ratios of MBA, depicted a decrease in drug loading from 70.46% to 58.81% (Figure 1d). Based on the results of CAP loading, CHF9 was the optimized formulation showing maximum loading (72.43%) of CAP.

### 2.2. Swelling Behavior

**Figure 2:**
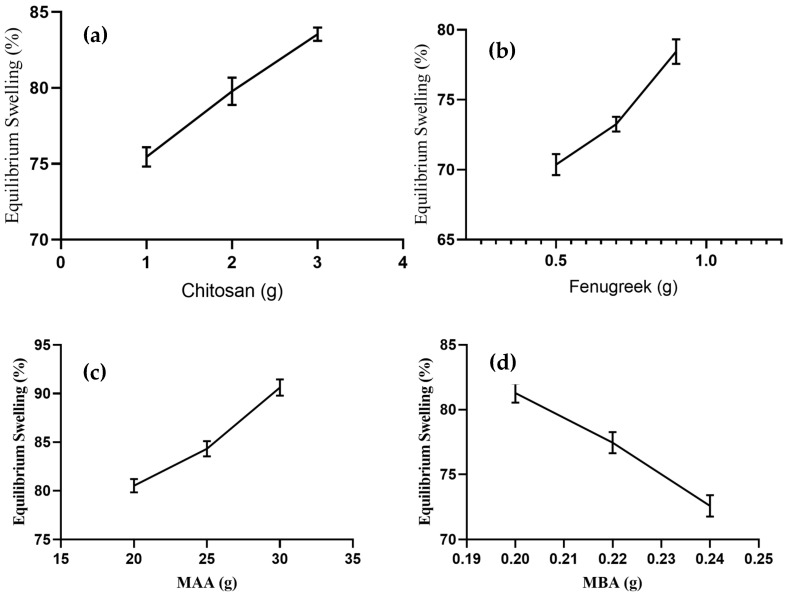
Effect of concentration of (**a**) chitosan, (**b**) Fenugreek, (**c**) MAA, and (**d**) MBA on equilibrium swelling.

Swelling response of formulations was noted in a phosphate buffer solution with pH 7.4. The effect of concentration of chitosan, fenugreek, MAA, and MBA on swelling response was determined, as presented in Figure 2.

Formulations of CHF1 to CHF3 were synthesized by varying proportions of chitosan, keeping quantities of other ingredients constant, and they revealed an increase in swelling percentage from 75.44–83.57% (Figure 2a) upon increasing chitosan concentration. Amino groups in the chitosan structure repel each other in an ionized state, resulting in the expansion of the network. This contributes to the penetration of more physiological fluid inside the hydrogels [13]. The swelling response was also promoted in the case of fenugreek gum-based formulations (CHF4-CHF6), and it ranged from 70.37–78.44% (Figure 2b). This increased swelling was attributed to deprotonation of hydroxyl groups from fenugreek and carboxylic groups from MAA, resulting in the development of electrostatic repulsive forces within these ionized groups [12].

An enhanced swelling rate (80.53–90.62%) was observed for formulations CHF7-CHF9, with variable concentrations of MAA (Figure 2c). Excessive carboxylic groups are available from monomers in the ionized state to bind with reactive sites of fenugreek and chitosan, resulting in volume expansion of the grafted system [14]. With the sequential rising of MBA in formulations CHF10-CHF12, the swelling response decreased from 81.28–72.58% (Figure 2d). The effect was attributed to higher polymerization by MBA, giving rise to reduced porosity and improved cross-linked density of the network. So, the penetration of fluid into the grafted network was reduced. Similar impacts of MBA on swelling response were reported previously in a study conducted by Malik et al. in 2020 [15].

### 2.3. Sol-Gel Fraction

**Figure 3:**
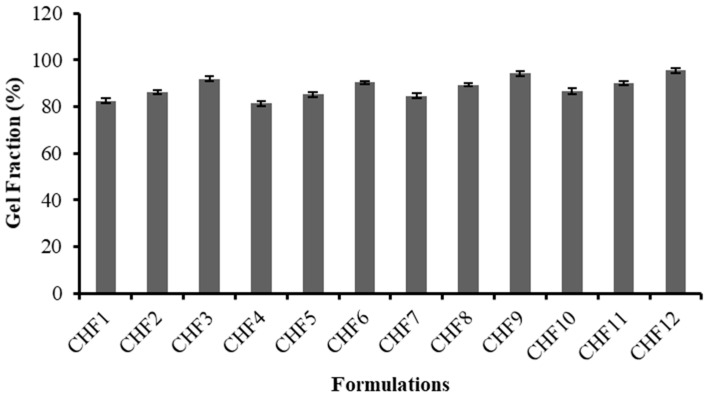
Gel fraction (%) of formulations CHF1-CHF12.

The chitosan/fenugreek-g-poly (MAA) carrier system was evaluated for sol and gel fraction to assess the reactant utilization during cross-linking reaction. All formulations exhibited gel fractions ranging from 81.42–95.51%, as presented in Figure 3.

For formulations containing variable concentrations of chitosan (CHF1 to CHF3) and fenugreek (CHF4-CHF6), an increase in gel fraction was revealed, ranging from 82.55% to 91.83% and 81.42% to 90.46%, respectively. A progressive rise in gel fraction up to 94.15% was observed for formulations (CHF7-CHF9) developed by the sequential rising of MAA amount, as methacrylic acid provides an excess of carboxylic groups that have an affinity to link with reactive sites on the polymeric backbone; hence, they facilitate a polymerization reaction resulting in elevation of gel fraction.

Likewise, promotion in gel fraction (86.56% to 95.51%) was also noted for formulations CHF10 to CHF12, having increasing trends of MBA. A cross-linking agent (MBA) can facilitate the cross-linking density of network structure due to rapid polymerization, thus increasing gel fraction. A previous study by Ganguly et al. (2018) reported a similar rise in gel fraction by increasing the MAA and MBA amounts in hydrogels, as revealed in our study [16].

### 2.4. FTIR Spectroscopy

**Figure 4:**
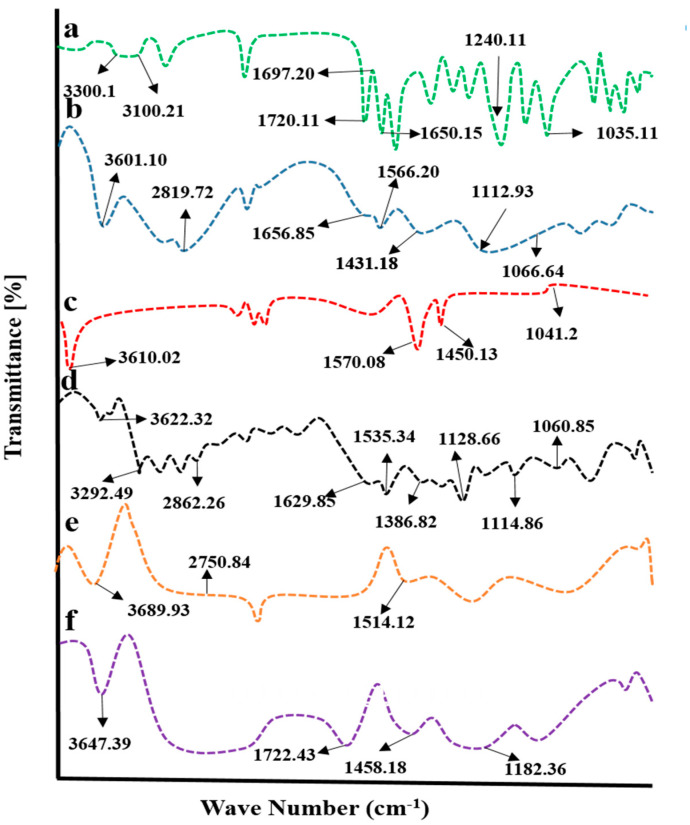
FTIR spectra of (**a**) capecitabine, (**b**) chitosan, (**c**) fenugreek extract, (**d**) physical mixture, (**e**) blank hydrogel, and (**f**) drug-loaded hydrogel.

Chitosan, fenugreek, and CAP were evaluated by FTIR spectroscopy individually and in a grafted network to ensure the compatibility of components. Results are displayed in Figure 4.

CAP displayed evident peaks at 1035.13 cm^−1^, 1240 cm^−1^, 1650.15 cm^−1^, 1697.20 cm^−1^, 1720.11 cm^−1^, 3100.21 cm^−1^, and 3300 cm^−1^, associated with stretching vibrations of C-F, tetrahydrofuran ring, pyrimidine carbonyl stretching vibrations, urethane carbonyl stretching vibrations, N-H stretching, and O-H stretching vibrations, respectively (Figure 4a).

IR spectrum of chitosan (Figure 4b) has revealed evident peaks at 1066.64 cm^−1^(CO stretching vibrations), 1112.93 cm^−1^ (COC asymmetric stretching vibrations), 1330.88 cm^−1^ (amide III peak), 1431.18 cm^−1^(OH bending vibrations), 1566.20 cm^−1^ (N-H bond bending vibration), 1656.85 cm^−1^ (N=C (imine bond)), 2879.72 cm^−1^ (asymmetric stretching vibrations of C-H), and 3601.10 cm^−1^ (overlapping of N-H and C-H stretching) [17].

Characteristic bands of fenugreek gum were displayed at 3610 cm^−1^, 1570.08 cm^−1^, 1450.13 cm^−1^, and 1041.22 cm^−1^ on the IR spectrum (Figure 4c) that was attributed to hydroxyl (-OH) groups stretching vibrations of sugar, polysaccharide skeleton vibrations, CH2 shear stretching, and ether group COC vibrations, respectively [18]. In the case of a physical mixture of components, prominent peaks of components were observed, showing no significant differences from pure components, as evident from the IR spectrum, thus proving the absence of physical interaction of ingredients.

In physical mixture, displacement of certain transmittance peaks of polymers was depicted with variation in intensities (Figure 4d). The characteristic peak of fenugreek gum appeared at 3610 cm^−1^ and was shifted to a higher wave number, i.e., 3689.93 cm^−1^, in the unloaded hydrogel. At the same time, chitosan-evident peaks presented at 1566.20 cm^−1^ and 2879.72 cm^−1^ due to the bending vibration of N-H bond and C-H asymmetric stretching vibrations were displaced to 1514.12 cm^−1^ and 2750.84 cm^−1^ on the IR spectrum of unloaded carrier system, showing a complexation of components within the network (Figure 4e).

The FTIR spectrum of CAP-loaded hydrogels showed displacement of prominent peaks of chitosan, fenugreek and CAP. CAP absorption peak due to N-H stretching presented at 1720.11 cm^−1^ was shifted to 1722 cm^−1^, evidencing complex formation and entrapment of CAP within the grafted network (Figure 4f).

### 2.5. Scanning Electron Microscopy

**Figure 5:**
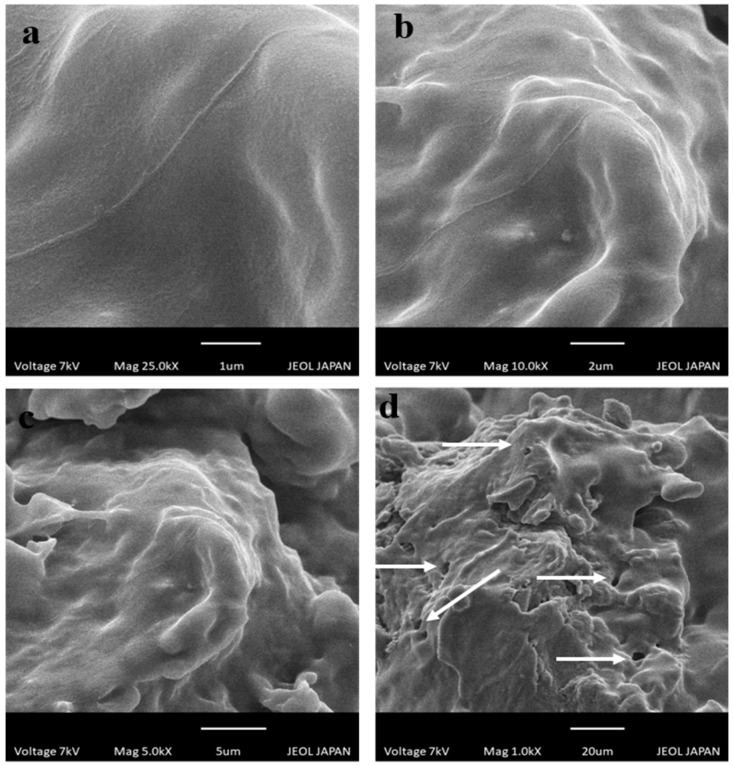
SEM photomicrographs of chitosan/fenugreek-g-poly (MAA) hydrogels at (**a**) 25 KX, (**b**) 10 KX, (**c**) 5 KX, and (**d**) 1 KX magnifications.

Scanning electron microscopy was conducted at 1 µm (Figure 5a), 2 µm (Figure 5b), 5 µm (Figure 5c), and 20 µm (Figure 5d) scale bar to study the surface morphology of developed drug-loaded hydrogels. Smooth surfaces were shown to have a glossy appearance. Hydrogels also exhibited pores on the surfaces, as revealed by SEM micrographs in Figure 5. Porosity in the grafted network is helpful in the absorption of biological fluid that causes swelling of hydrogel and release of CAP from the polymeric network [19].

### 2.6. Thermal Analysis

**Figure 6:**
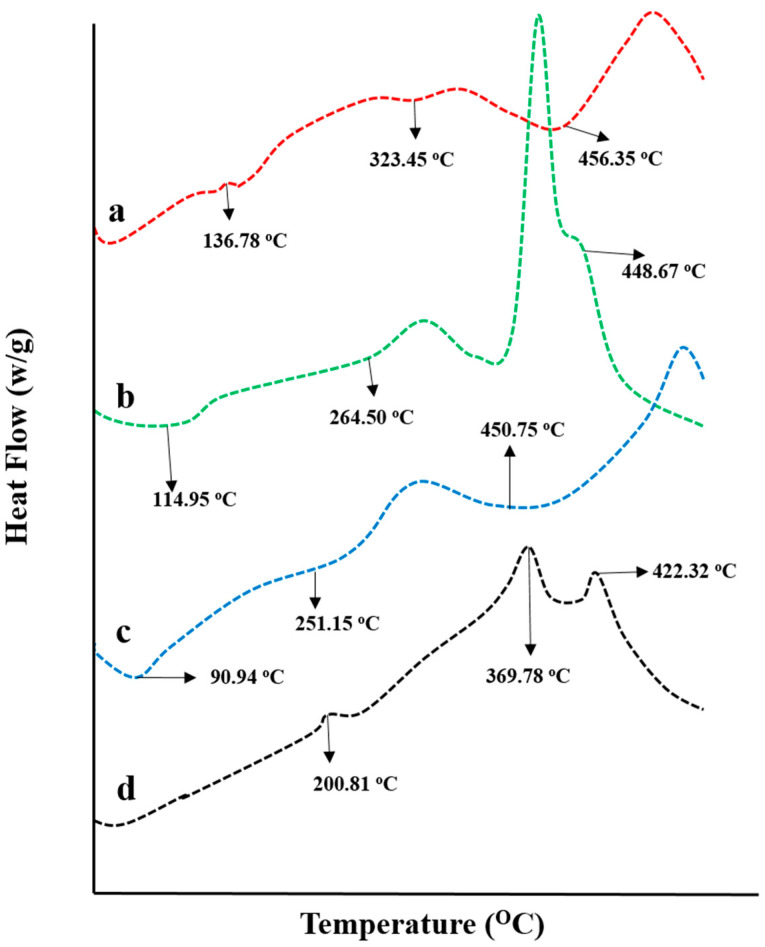
DSC thermograms of (**a**) capecitabine, (**b**) fenugreek extract, (**c**) chitosan, and (**d**) drug-loaded hydrogel.

The thermal behavior of pure chitosan, fenugreek gum, and CAP was investigated individually and in cross-linked structure by conducting DSC-TGA studies. DSC spectra are presented in Figure 6. CAP DSC spectrum has shown an endothermic peak at 132.31 °C (0.02265 J/g), corresponding to its melting range, confirming its crystalline nature. Another peak presented at 159.70 °C (0.004427 J/g) was describing the partial degradation of CAP, while the peak at 456.35 °C (0.0007347 J/g) was attributed to its complete combustion (Figure 6a).

DSC spectrum of fenugreek gum presented an endothermic peak at 114.95 °C (0.006124 J/g), showing moisture removal from the gum. Two more peaks recorded at 264.50 °C (0.03039 J/g) and 379.46 °C (0.1580 J/g) depict the polymer’s melting range and decomposition, respectively. The entire combustion of fenugreek gum was noticed at 448.67 °C (0.0005067 J/g) (Figure 6b). Similarly, in the DSC thermogram of chitosan showed endothermic peaks at 90.94 °C and 251.15 °C due to water removal and melting of polymer, respectively. While peak displayed at 450.75 °C represented the entire degradation of the polymer (Figure 6c).

DSC spectrum of CAP loaded chitosan/fenugreek-g-poly (MAA) network presented the transformation of melting range peaks of CAP and chitosan at 132.31 °C and 251.15 °C to higher temperatures, i.e., 200.81 °C and 358.06 °C, thus evidencing the stability of hydrogels at higher temperatures (Figure 6d). Moreover, the existence of another peak was seen at 407.93 °C, with 0.05106 J/g of energy demonstrating the partial combustion of the grafted system. These results proved the stability and fabrication of the newly developed network system. Stability of newly developed chitosan-based hydrogels was also proved by DSC analysis conducted by Lino et al. (2017) [20].

**Figure 7:**
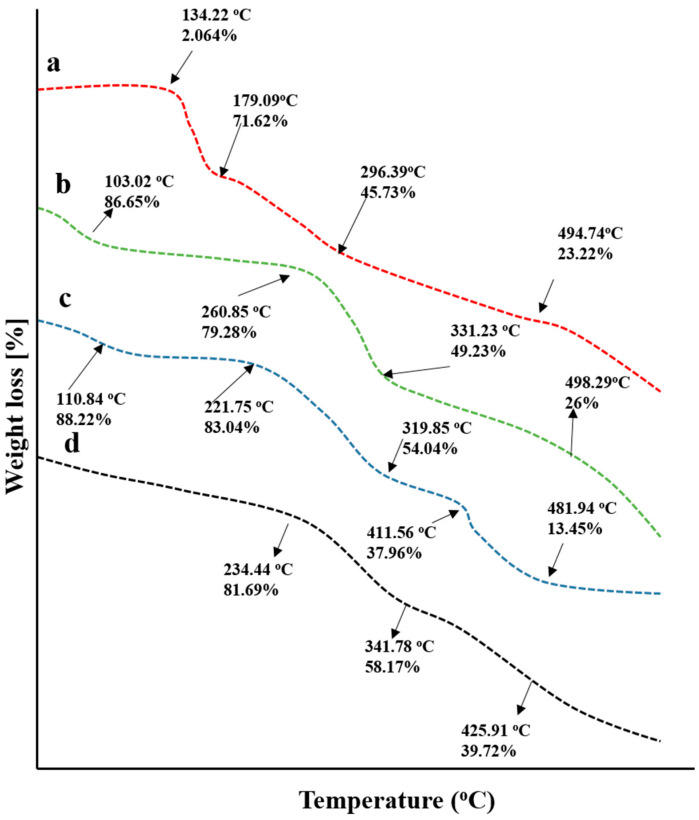
TGA thermograms of (**a**) capecitabine, (**b**) chitosan, (**c**) fenugreek seed extract, and (**d**) drug loaded hydrogels.

Weight loss stages with a subsequent rise in temperatures were recorded by conducting a TGA study, as shown in Figure 7. TGA thermogram of chitosan presented 13.35% mass loss at 103.02 °C that was increased gradually to 20.72% at 260.85 °C, 50.77% at 331.23 °C, and 74% at 498.29 °C that might be the result of polymer decomposition.

In the case of fenugreek gum, an initial weight loss of 11.78% occurred at 110.84 °C that was increased to 16.94% at 221.75 °C, describing the removal of water content associated with the gum. The next degradation stages were recorded at 319.85 °C and 411.56 °C with 45.87% and 62.04% loss in masses, respectively. The final stage was observed with 86.55% mass loss at 481.94 °C, describing the entire decomposition of the polymer.

For drug-loaded grafted networks, 19% weight loss was recorded at 234.55 °C, which was increased to 41.83% at 341.78 °C. The final decomposition of the grafted network took place at 425.91 °C and revealed 60.28% of weight loss. Chitosan/fenugreek-g-poly (MAA) hydrogels exhibited good stability, as a percentage of mass remaining was more than individual components, i.e., fenugreek, chitosan, and CAP at higher temperatures. Similar findings were reported previously by a study conducted by Bal and Swain 2019 on a grafted copolymer of fenugreek seed mucilage [21].

### 2.7. XRD Analysis

**Figure 8:**
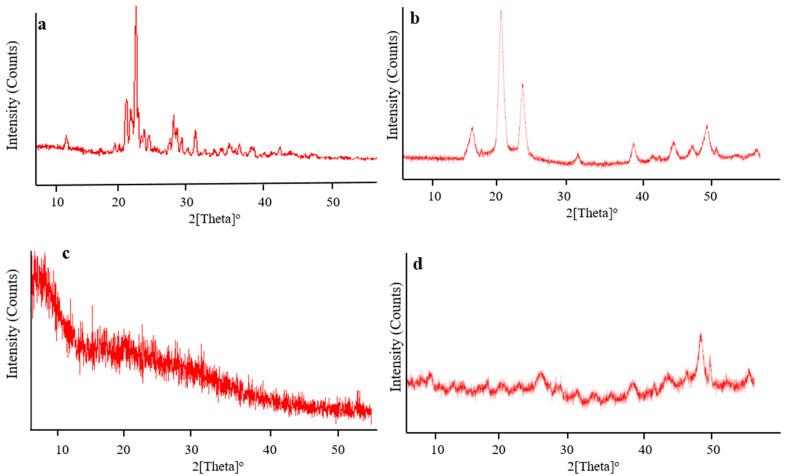
XRD diffractograms of (**a**) CAP, (**b**) chitosan, (**c**) fenugreek gum and, (**d**) drug-loaded hydrogels.

The amorphous or crystalline character of pure chitosan, fenugreek, CAP, and drug-loaded hydrogels were evaluated by recording XRD diffractograms, as shown in Figure 8. Sharp and intense peaks of CAP were revealed at 10.75°, 18.95°, 19.6°, 20.05°, 21.45°, 22.1°, 25.45°, and 28.65° (Figure 8a), while characteristic peaks of chitosan were presented at 17.35°, 21.15°, 24.49°, 32.65°, 39.87°, 45.02°, 47.53°, and 50.16° (Figure 8b), representing their crystalline nature.

XRD diffractogram of fenugreek (Figure 8c) displayed no sharp peaks, while fused curves proved the amorphous nature of the gum. In the case of a polymerized network, characteristic peaks of chitosan observed at 17.35°, 21.15°, and 24.49° (Figure 8d) were markedly reduced in intensity, reflecting its polymerization and conversion into less crystalline nature in the grafted network. The reduction in crystallinity of the grafted network might be associated with cross-linking of chitosan with the monomer (MAA) carried out by MBA. This resulted in the fabrication of hydrogels having a higher amorphous form. Moreover, sharp peaks of CAP were absent on the diffractogram of drug-loaded hydrogels, hence evidencing its amorphous dispersion into the polymerized structure. Khan and Ranjha (2014) also reported the amorphous dispersion of the drug within the chitosan-based hydrogels by conducting XRD analysis [22].

### 2.8. Elemental Dispersive X-ray Spectroscopy

**Figure 9:**
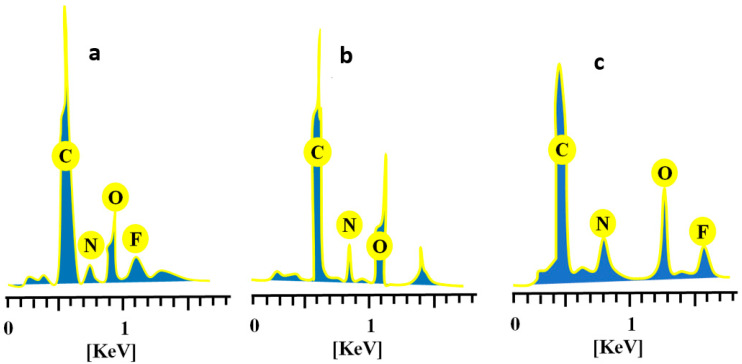
EDX spectra of (**a**) CAP, (**b**) unloaded, and (**c**) CAP-loaded chitosan/fenugreek-g-poly (MAA) hydrogels.

Pure CAP, unloaded, and drug-loaded hydrogels were evaluated for elemental composition by performing elemental dispersive spectroscopy. EDX spectra and percentage composition of elements for each case are displayed in Figure 9 and Table 1, respectively. Specific concentrations of carbon, nitrogen, oxygen, and fluorine (56.06%, 10.52%, 29.54% and 3.87%) were indicated by quantitative data on CAP (Figure 9a). Similarly, these elements were also identified in drug-loaded chitosan/fenugreek-g-poly (MAA) hydrogels in concentrations of 51.40%, 1.24%, 38.04%, and 9.32%, as observed in Figure 9c. In the case of unloaded hydrogels, the peak of fluorine was missing, as it is only an essential element of CAP, while carbon, nitrogen and oxygen were displayed in concentrations of 47.88%, 4.14% and 47.99%, respectively (Figure 9b).

### 2.9. In-Vitro CAP Release Studies

**Figure 10:**
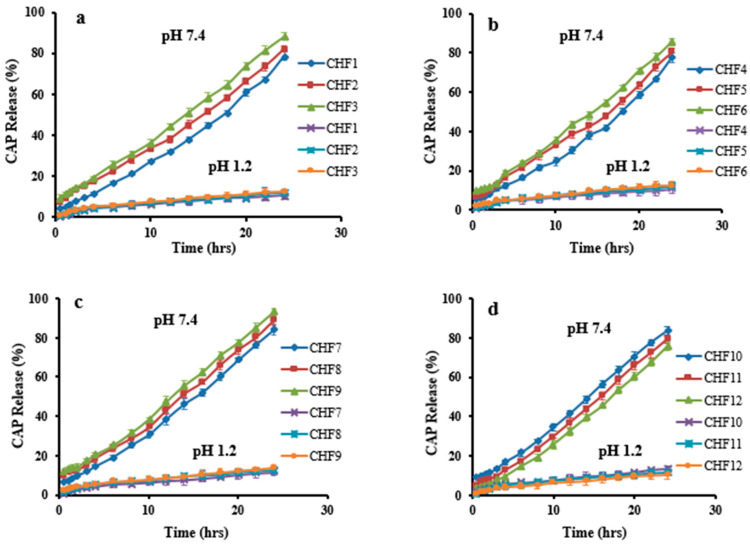
CAP release profile at pH 1.2 and pH 7.4. Effect of concentration of (**a**) chitosan, (**b**) fenugreek, (**c**) MAA, and (**d**) MBA on CAP release.

Dissolution studies of the developed formulations (CHF1-CHF12) were conducted in acidic and basic pH to investigate CAP in vitro release rate. Low drug release of 10.47% to 13.65% was revealed at pH 1.2. However, it was enhanced significantly (*p* < 0.05) at pH 7.4 that was ranged within 75.81–93%, as presented in Figure 10. Release of the drug was markedly affected by the sequential rising of chitosan, fenugreek, MAA, and MBA contents in formulations CHF1-CHF3, CHF4-CHF6, CHF7-CHF9, and CHF10-CHF12, respectively.

CAP release was increased from 78.55–88.11% (Figure 10a), with an increasing chitosan ratio, and this effect is attributed to the ionized state of the amino group of chitosan, creating repulsive forces and expansion of the network. So, excess fluid can penetrate inside the hydrogel resulting in more loading and release of CAP [14]. Similarly, the release rate was enhanced from 77.54–85.41% by a sequential rising in fenugreek gum content (Figure 10b).

A significant increase in CAP release was noticed (84.14–93%) for formulations having increasing amounts of MAA (Figure 10c). At pH 7.4, the availability of carboxylic groups from MAA in an ionized state creates repulsion and expansion within the grafted system, thus allowing penetration of excessive physiological fluid inside the network. As a result, a higher release of CAP resulted from hydrogels.

Unlikely, the release of CAP decreased from 83.29–75.81% by increasing the MBA amounts in formulations CHF10-CHF12 (Figure 10d) due to the promotion of the network’s cross-linking density by the cross-linking agent. Therefore, reduced penetration of physiological fluid into hydrogel resulted in less release of CAP. These impacts of MBA on drug release were in agreement with the previous work of Abdullah et al. (2018) [23].

By applying kinetic models on release data, values of the regression coefficient (R^2^) revealed that all formulations followed zero-order kinetics, proving CAP’s controlled release pattern. R^2^ values ranged between 0.9700–0.9936, as documented in Table 2. Based on the exponent ‘n’ value, it can be observed that most of the formulations depicted super case II transport. While formulations CHF3 and CHF9 followed non-Fickian diffusion because values of n were 0.851 and 0.846, respectively, for these formulations.

Based on the results of the previous studies, CHF9 showing maximum loading (72.43%) of CAP was selected for further studies.

### 2.10. Toxicity Studies

Toxicity studies were performed on healthy rabbits to ensure the safety of the newly developed carrier system. The study carefully monitored various parameters, such as body weight, water and food intake, fever, diarrhea, and ocular and dermal toxicity. Significant variations in body weight, water, and food consumption were not seen in the control and tested groups, as presented in Table 3. No other signs of illness were observed in animals of both groups. Hematological and biochemical analysis of blood samples displayed acceptable results, as shown in Table 4 and Table 5. The value of white blood cells was comparatively high in group B, as it was administered with the hydrogels that might have elevated the white blood cells but lying within the official limits.

**Figure 11:**
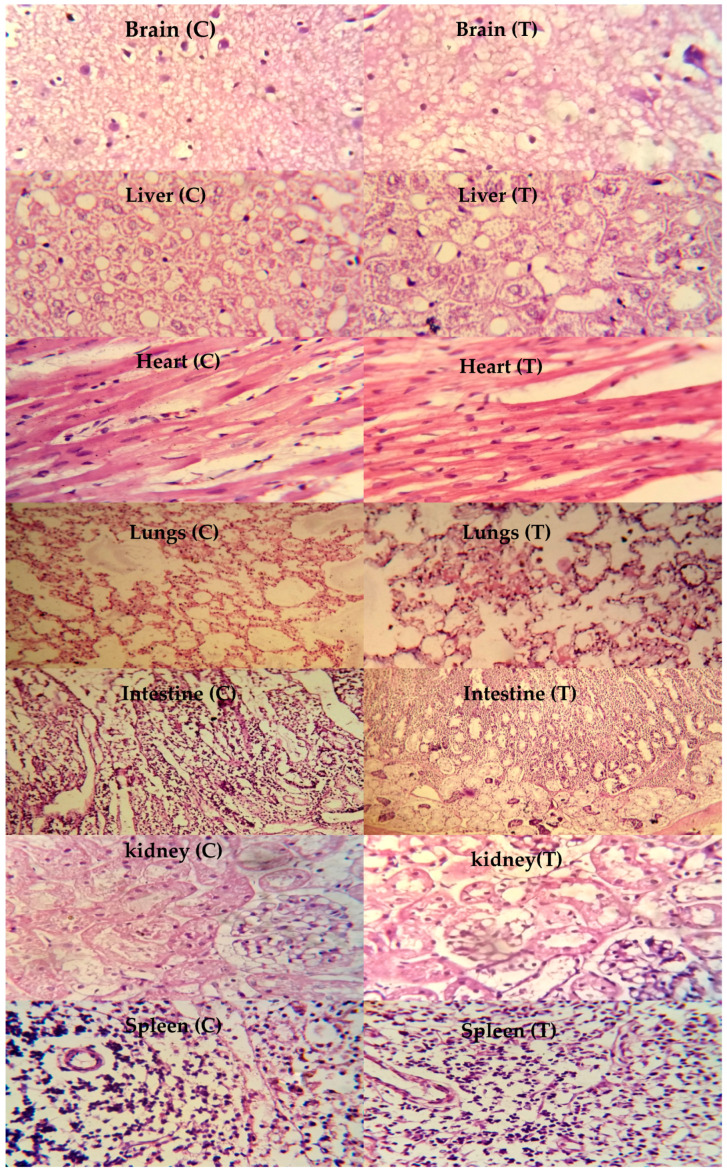
Histopathological examination of rabbit’s vital organs, C: control group and T: treated group, magnification ×40.

The toxicity of the chitosan/fenugreek-g-poly (MAA) network was investigated by histopathological examination of vital organs. Animals were sacrificed on the 14th day of the study; vital organs were removed and stored in containers containing 10% formalin solution. Histological examination was performed microscopically by preparing H&E-stained tissue slides of these organs. As presented in Figure 11, no signs of degeneration, lesions, or other ailments were observed in tissues of vital organs (control and treated groups).

Photomicrographs of the brain showed normal axons with nuclei, intact cortical regions displaying no cellular degeneration, and inflammatory cell infiltration. In the liver section of both groups, slight hyperplasia and inflammatory cell accumulation were noticed. Cardiac tissues displayed a precise pattern of cardiomyocytes without any sign of hypertrophic cells and myocardial infarction. Microscopic examination of lung tissues displayed slight edema and alveolar accumulation in both groups, but lung fibrosis was not observed.

The intestines of animals were observed to be normal, with no evidence of inflammation. Intact muscularis and normal columnar epithelium were visible without any sign of injury. Microscopic evaluation of kidneys showed intact glomerulus, Bowman capsule, and normal tubules. Moreover, a normal spleen was evident with uniform distribution of white blood cells in the white pulp. There was no significant difference between the control group and the treated group with chitosan/fenugreek-g-poly (MAA) hydrogels. Batool et al. 2022 developed hydroxypropyl-β-cyclodextrin based hydrogels for cytarabine delivery and performed toxicity studies in rabbits. They reported quite similar findings, as observed in our work [24].

### 2.11. In Vivo Pharmacokinetic Studies

**Figure 12:**
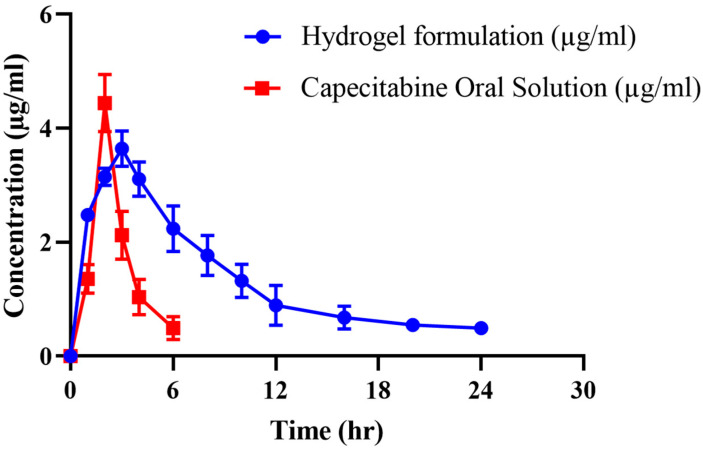
Plasma concentration vs. time profile of CAP after administration of oral solution and hydrogels.

Pharmacokinetic experiments were carried out in healthy rabbits to further investigate CAP’s sustained release response from the grafted network. For this purpose, both groups of animals (B and C) were given an oral solution of pure CAP and optimized hydrogel formulation (CHF9) at equivalent doses, followed by 5–10 mL of water. Values of pharmacokinetic parameters are presented in Table 6, while a comparison of the CAP plasma profile of both formulations is displayed in Figure 12. Drug concentrations in plasma were detected at specified time intervals. In the case of oral solution, the maximum concentration of CAP (C_max_) was noted to be 4.44 μg/mL, achieved within 2 h due to rapid absorption of the drug. These parameters were found to be 3.64 μg/mL and 3 h for hydrogel formulation, respectively. The drug’s half-life was 1.27 h, which was prolonged to 13 h when administered as chitosan/fenugreek-g-poly (MAA) hydrogels. That extended half-life can be explained in terms of the slow release of CAP from polymeric matrix resulting in its sustained effect in plasma and increased bioavailability. Moreover, a significant difference was also revealed within other pharmacokinetic parameters in the case of pure drug and cross-linked network, i.e., MRT of the pure drug (3.02 h) was prolonged to 16.09 h, and AUC was extended from 10.87 µg h/mL to 42.88 µg h/mL. Hence, the chitosan/fenugreek-g-poly (MAA) network system proved to be a suitable and efficient platform for delivering and prolonging the release of CAP and other chemotherapeutic agents, as confirmed by in vivo studies. Hassan et al. (2022) developed chitosan nanoparticles to improve olmesartan medoxomil bioavailability through the nasal route and performed pharmacokinetic studies in rats. They reported a 11.3-fold increase in bioavailability of the drug when administered in the form of nanoparticles, as proved by greater AUC values [25]. Our work on hydrogels also displayed improvement in the pharmacokinetic parameters of drug. 

## 3. Conclusions

In the current work, smart pH responsive chitosan/fenugreek-g-poly (MAA) network system was successfully fabricated and loaded with capecitabine for its controlled delivery. The developed hydrogels were evaluated for different characteristics, including drug loading percentage, swelling response, structural and compositional features, thermal stability, elemental analysis, morphology, and drug release kinetics. All formulations revealed the pH responsive character as significantly higher swelling and drug release were observed at alkaline pH compared with an acidic one. Optimum results were exhibited by the formulation of CHF9, showing the highest drug loading, swelling, and CAP release. Optimized formulation was further tested for its safety profile by carrying oral toxicity studies in healthy rabbits showing no ocular and dermal toxicity, pathological change in the blood, or other abnormalities. Moreover, in vivo experiments were also conducted on rabbits. The results of pharmacokinetic parameters displayed prolonged half-life, AUC, and MRT of CAP after administration of chitosan/fenugreek-g-poly (MAA) hydrogels, hence evidencing the sustained release profile of the drug from the grafted network. So, the developed carrier system exhibited excellent potential for delivering CAP and other drugs at a controlled rate.

## 4. Materials and Methods

### 4.1. Materials

CAP was purchased from Wuhan vanz Pharma, Jingkai Future city Hubei, Wuhan, China. Chitosan, methacrylic acid (MAA) (99%), N,N-methylene bisacrylamide (MBA) (99%), ammonium persulfate (APS) (99%), and methanol (99.7%) were purchased from Sigma-Aldrich Co., St Louis, MO, USA. Fenugreek seeds were purchased from the local market. Ethanol, N-hexane, potassium dihydrogen phosphate, orthophosphoric acid, and sodium hydroxide were purchased from Dae-Jung, Korea. All the chemicals used were of analytical grade.

### 4.2. Preparation of Fenugreek Seed Mucilage Powder

Fenugreek seeds were immersed in distilled water (1:10 *w/v*) and placed in an oven at 40 °C for 4–6 h. After complete soaking, the seed mixture was filtered through a muslin cloth to obtain the filtrate free from the fiber contents. The resulting viscous solution was washed with n-hexane for 20 min, poured into petri dishes, and placed in an oven for drying at 40–45 °C. Dried gum was ground and passed through sieves to obtain fine powder [10].

### 4.3. Synthesis of Chitosan/Fenugreek-g-poly (MAA) Hydrogels

**Figure 13:**
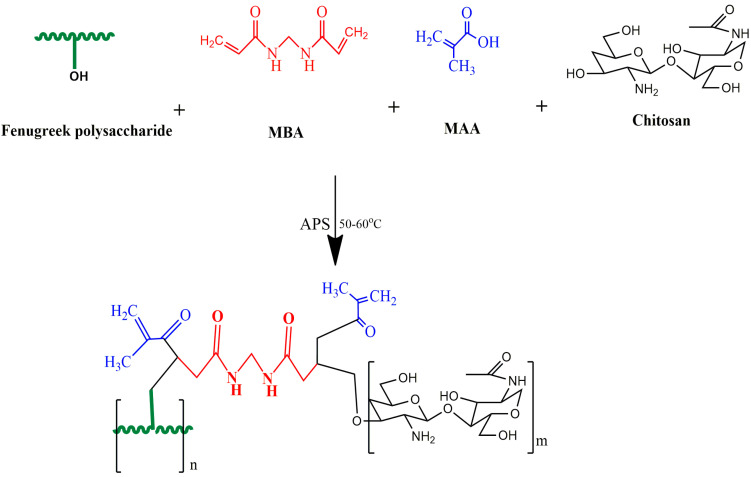
Proposed schematic diagram of synthesis of chitosan/fenugreek-g-poly (MAA) hydrogels.

The free radical polymerization technique was employed to synthesize chitosan/fenugreek-g-poly (MAA) hydrogels [26]. Twelve formulations were developed (CHF1-CHF12), using various proportions of polymers, monomers, and cross-linking agents, as presented in Table 7. Chitosan and fenugreek seed powder were dissolved in acetic acid solution (1% *v/v*) and distilled water, respectively, using a magnetic stirrer (VELP Scientifica, Milano, Italy) at 110 rpm. After completely dissolving, both polymer solutions were mixed with stirring (120 rpm). APS solution was prepared separately in distilled water followed by addition to the polymer solution with thorough agitation. MAA (monomer) was heated slightly with stirring, then MBA (as a cross-linker) was added to it, and stirring was continued until the solution became clear. The MAA/MBA solution was poured into the polymer mixture, and the final volume was adjusted by the addition of distilled water. The prepared mixture was divided into glass tubes, covered with aluminum foil, and sonicated for 5 min in a digital water bath (WB10-RoHS, VWR International, LLC, Radnor, PA, USA) at 65 °C for 24 h to allow polymerization. Upon solidification, test tubes were taken off of the water bath and then broken carefully to remove the hydrogels, which were divided into small discs, and then they were washed with ethanol and water solution (70:30) for 30 min to remove unreacted contents. Discs were dried in an oven at 45 °C and then at room temperature [26]. The schematic presentation and proposed chemical structure of the resultant carrier system is presented in Figure 13.

### 4.4. Characterization

#### 4.4.1. Loading of CAP

1% *w/v* CAP solution was prepared in phosphate buffer (pH 7.4) with the aid of magnetic stirring for 30 min at room temperature. To achieve drug loading, the pre-weighed discs were immersed in the CAP solution till equilibrium. After that, the swollen discs were taken out from the solution and excess solution was swabbed using filter paper, followed by drying in a hot air oven (Memmert, Schwabach, Germany) at 45 °C and then at room temperature [27].

#### 4.4.2. Quantification of CAP

The CAP loading (%) was calculated as follows

(1) $$\mathrm{Drug}\;\mathrm{loading}\left(\%\right)=\frac{\mathrm{WD}-\mathrm{Wd}}{\mathrm{Wd}}\times 100$$

where WD = final weight of the hydrogels discs.

Wd = initial weight of dried hydrogel discs.

#### 4.4.3. In-Vitro Swelling Studies

Grafted network was evaluated for its pH responsiveness by conducting swelling studies where the dried discs, after weighing on an electric weighing balance (Shimadzu, AUW220D, Kyoto, Japan), were inserted in a phosphate buffer (pH 7.4) at 37 °C. For a defined time duration (up to 48 h), the discs were taken off of the buffer, swabbed, and reweighed. The experiment was carried out until all the discs attained equilibrium. The percentage swelling of the hydrogels was calculated as follows [28].

(2) $$\mathrm{Swelling}\;\left(\%\right)=\frac{\mathrm{Wt}-\mathrm{Wo}}{\mathrm{Wo}}\times 100$$

where, Wo and Wt are the initial weights of the dried disc and the swollen disc at time t, respectively.

#### 4.4.4. Sol-Gel Fraction

Sol and gel fraction was calculated to identify the amounts of cross-linked and uncross-linked part of the chitosan/fenugreek-based network. For this, pre-weighed dried discs were pulverized into small pieces. Extraction was carried out for 4 h using a Soxhlet apparatus containing boiled distilled water so that the unreacted reactants could be removed from the co-polymeric network. Filter paper was used to remove the extracted hydrogel pieces, which were then dried for 24 h at room temperature, then in a hot air oven at 40–45 °C, before being reweighed [29]. Sol and gel fraction (%) were calculated as follows:

(3) $$\mathrm{Sol}-\mathrm{fraction}\left(\%\right)=\frac{\mathrm{Wo}-\mathrm{Wt}}{\mathrm{Wo}}\times 100$$

(4) Gel−fraction=100−sol−fraction

where Wo = Initial weight of dried disc before extraction and Wt = Final weight of the dried disc after extraction.

#### 4.4.5. Fourier-Transforms Infrared (FTIR) Spectroscopy

The FTIR examination of pure CAP, chitosan, fenugreek gum, a mixture of polymers, monomer, cross-linker, and CAP-loaded and unloaded hydrogels was performed to analyze the complexity and compatibility of the mixture. All the samples were mixed with potassium bromide and compressed into discs of 12 mm thickness using 65 kN pressure for 1 min. Samples were scanned at 4000 to 500 cm^−1^ using a Thermos Fischer scientific Nicolet6700TM FTIR spectrophotometer (Waltham, MA, USA) [30].

#### 4.4.6. Scanning Electron Microscopy (SEM)

SEM was used to examine the surface morphology and shape of the prepared hydrogels (JSM-5910 instrument, JEOL, Akishima, Japan). The dried discs of hydrogel were adhered on an aluminum stub double adhesive tape, followed by the application of a gold coating of thickness 300 Å on the aluminum stubs and scanned at a current of 10 kV [31].

#### 4.4.7. Differential Scanning Calorimeter (DSC)

DSC studies were also performed on drug, polymers, monomers, and newly developed hydrogels to ascertain the thermal nature. Samples in powder form were placed in an aluminum DSC pan in hermetically sealed conditions and thermal analyzer SDT (Q600, TA instrument, Waters ^TM^, New Castle, DE, USA) was operated at 0–400 °C over a heating rate of 10 °C/min with continuous flow of nitrogen (100 mL min) [32].

#### 4.4.8. Thermogravimetric Analysis (TGA)

TGA thermal analysis was performed using the same conditions of the DSC to compare the newly developed polymeric network’s thermal stability to the formulation’s ingredients at high temperatures.

#### 4.4.9. Powder X-ray Diffraction (PXRD) Analysis

Pure ingredients and the CAP-loaded network were evaluated for the confirmation of their amorphous or crystalline nature by performing powder X-ray diffraction studies. Analysis was carried out by applying 40 kV energy and 28 mA current in X-ray analytical Xpert powder diffractometer (PANalytical, Almelo, The Netherlands). Scanning range was set at 2θ from 10–70° for recording diffractograms [33].

#### 4.4.10. Elemental Dispersive X-ray Spectroscopy (EDX)

Microanalysis technique was employed to determine the elemental composition and atomic weight of components using INCA 200 m Oxford, UK. The technique was used to analyze both the unloaded and loaded pure drug hydrogels, and the spectra were recorded [34].

#### 4.4.11. In-Vitro CAP Release

Dissolution experiments were performed to determine the release profile of CAP from hydrogels using the USP dissolution apparatus Type-II (USP dissolution (Sr8plus Dissolution Test Station, Hanson Research, Chatsworth, CA, USA)), with 900 mL of buffer solution at 37 ± 0.5 °C and a paddle speed of 50 rpm. Samples were taken at predetermined intervals, including 0.5, 1, 1.5, 2, 3, 4, 6, 8, 10, 12, 14, 16, 18, 20, 22, and 24 h, and CAP was quantitatively analyzed at a wavelength of no greater than 300 nm using a UV visible spectrophotometer [35,36].

#### 4.4.12. Kinetic Modeling on CAP Release Data

CAP release data were processed by applying different kinetic models (zero order, first order, Higuchi and Korsemeyer-Peppas) through DD solver to confirm their mode of release. Best fit model and mechanism of drug release were confirmed by values of R^2^ and n, respectively. The value of *n* = 0.45 described the release following the Fickian diffusion mechanism. n ranging within 0.45–0.89 represented the anomalous transport and 0.45 < *n* > 0.89 showing the Case II transport (zero-order kinetics) [37].

Zero-order kinetics

(5) Ft = Fo−Kot

First-order kinetics

(6) $$\ln\left(1-F\right)=-K_{1}t\;$$

Higuchi model

(7) $$\mathrm{Ft}=K_{H}t^{1/2}$$

where Ft = Fraction of drug release in time t, F_o_ = Total conc. of CAP in grafted networks, K_o_ K_1_, and K_H_ are the rate constants for Zero order, First order, and Higuchi models, respectively.

Korsmeyer-Peppas model

(8) $$\frac{\mathrm{Mt}}{M^{\propto }}={\;k}_{\mathrm{kp}}t^{n}$$

M_t_/M_∞_ describes the amount of CAP released at time t, K_kp_ is rate constant, and n shows the release exponent.

### 4.5. Acute Oral Toxicity Studies

Twelve healthy albino rabbits were used to determine the acute oral toxicity of the chitosan/fenugreek-g-poly (MAA) hydrogel in accordance with the guidelines of OECD. The study protocols were reviewed and approved by the Institutional Research Ethics Committee of Faculty of Pharmacy, The University of Lahore, notification no. IREC-2019-137A. The test animals were acclimated in stainless steel cages for seven days while being fed a healthy diet and given access to water. Rabbits were equally divided into two, i.e., group A (control) and group B (treated). Following the overnight fasting, each animal in group B received pulverized hydrogel (2 g/kg dose) via a feeding tube. Pulverized hydrogel particles were obtained by crushing hydrogel discs in pestle and mortar followed by their sieving through sieve no. 10 in order to obtain uniform size hydrogel particles. Both groups were then monitored for 14 days for changes in body weight, food and water intake, skin allergies, and physical changes. On the seventh day, the animals’ ears were cleaned off (hair was removed), and 2–3 mL of the blood sample was collected from the marginal vein of the ear by using 3 cc syringes (Injekt^®^). Following transferring into EDTA tubes, these samples were subjected to centrifugation for 15 min at 5000 rpm (Hitachi Zentrifugen EBA 20, Hitachi Ltd., Tokyo, Japan). Samples were analyzed for AST, ALT levels, hematological examination, and lipid and renal profiles. On the 14th day, animals were injected with anesthetic agents, i.e., ketamine and xylazine (70:30) at 1 mL/kg dose and slaughtered to remove the vital organs (brain, heart, liver, intestine, lungs, spleen, and stomach). These were washed and then stored in separate containers having 10% formalin solution. Histopathological analysis was conducted after preparing the slides of all vital organs of both groups [38].

### 4.6. Pharmacokinetics Study

In vivo studies were conducted in healthy male rabbits for pharmacokinetic evaluation of CAP from the grafted network. For this purpose, eighteen rabbits (2–2.5 kg) were acclimatized under a 12/12 h dark/light cycle for one week. Animals were then divided into three groups (*n* = 6). Group A was considered the control group, while groups B and C were the tested groups, where animals fasted overnight before the experiment with free access to water. Pure CAP solution in 10 mg/kg dose was given to group B, while CAP-loaded hydrogels (crushed form) in a similar dose were filled in hard gelatin capsules and administered to group C with 5–10 mL of water. At predetermined time intervals after dose administration, 2–3 mL of blood samples were withdrawn from the jugular vein and collected in EDTA tubes immediately. Tubes were then centrifuged for 10 min at 5000 rpm for plasma separation. Each plasma sample was deproteinized, adding an equal volume of HPLC-grade methanol. After vortex mixing for 3 min, tubes were subjected to further centrifugation for 15 min, and the supernatant was collected for quantitative analysis of CAP after diluting with the mobile phase. Animals were given a washout period of two weeks. After this, pure CAP solution was administered to group C, while drug-loaded hydrogels were given to group B in a similar dose. The experiment was repeated, as described above. Analysis of CAP in samples was carried out using a developed HPLC method with certain modifications [39]. Water and acetonitrile mixture (50:50 *v/v*) was used as mobile phase at a flow rate of 1 mL min. A reverse phase C18 column was used, having dimensions of 250 mm 4.6 mm 5 μ. CAP was detected by scanning at λ_max_ of 310 nm after transferring the samples into auto-sampler vials. Different pharmacokinetic parameters (C_max_, T_max_, MRT, t_1/2_, AUC_0-t_, AUMC_0-inf_ and Cl) were calculated using a non-compartmental analysis of the pharmacokinetic model.

## Figures and Tables

**Table 1:** Elemental composition of the drug, unloaded hydrogels, and drug-loaded hydrogels.

Materials	Elements	Weight %
CAP	C	56.06
	N	10.52
	O	29.54
	F	3.87
Unloaded chitosan/fenugreek-g-poly(MAA) hydrogels	C	47.88
	N	4.14
	O	47.99
Loaded chitosan/fenugreek-g-poly(MAA) hydrogels	C	51.40
	N	1.24
	O	38.04
	F	9.32

**Table 2:** Results of kinetic modeling on CAP release profile.

Formulation	Zero Order	1st Order	Higuchi	Korsmeyer-Peppas	
	R^2^	R^2^	R^2^	R^2^	n
CHF1	0.9847	0.9349	0.8149	0.9887	1.133
CHF2	0.9821	0.9571	0.8849	0.9812	0.875
CHF3	0.9746	0.9478	0.8928	0.9828	0.851
CHF4	0.9772	0.9277	0.8118	0.9804	1.132
CHF5	0.9852	0.9557	0.8690	0.9858	0.934
CHF6	0.9813	0.9527	0.8832	0.9855	0.887
CHF7	0.9903	0.9412	0.8439	0.9899	1.027
CHF8	0.9789	0.9397	0.8748	0.9811	0.904
CHF9	0.9700	0.9373	0.8910	0.9788	0.846
CHF10	0.9843	0.9516	0.8746	0.9862	0.915
CHF11	0.9936	0.9484	0.8359	0.9943	1.059
CHF12	0.9884	0.9372	0.8014	0.9971	1.186

**Table 3:** Clinical findings during acute oral toxicity studies.

Observations	Group A (Control)	Group B (Treated)
Signs of Illness	Not Observed	Not Observed
Body weight (Kg)		
Pretreatment	1.84 ± 0.03	1.96 ± 0.02
Day 1	1.84 ± 0.02	1.96 ± 0.03
Day 7	1.86 ± 0.04	1.97 ± 0.04
Day 14	1.87 ± 0.05	2.02 ± 0.02
Water intake (ml)		
Pretreatment	166.18 ± 1.14	179.15 ± 0.22
Day 1	174.85 ± 1.14	186.22 ± 1.44
Day 7	192.17 ± 3.21	200.43 ± 1.17
Day 14	202.43 ± 3.53	205.2 ± 3.64
Food intake (g)		
Pretreatment	72.25 ± 1.51	71.67 ± 1.08
Day 1	73.45 ± 1.10	74.31 ± 1.17
Day 7	75.31 ± 1.62	73.61 ± 1.43
Day 14	77.55 ± 1.19	75.28 ± 1.02
Dermal toxicity	Not seen	Not seen
Ocular toxicity	Absent	Absent
Mortality	Nil	Nil

Results represent the mean ± S.D.

**Table 4:** Results of hematological analysis of rabbits’ blood.

Parameters	Group A (Control)	Group B (Treated)
Hemoglobin (g/dL)	11.25	12.50
pH	7.13 ± 0.23	7.17 ± 0.22
White blood cells (×10^9^ L^−1^)	5.4 ± 0.42	6.5 ± 0.45
Red blood cells (×10^6^/µL)	4.94 ± 1.05	4.99 ± 1.35
Platelets (×10^9^ L^−1^)	4.72 ± 0.15	4.68 ± 0.41
Monocytes (%)	3.63 ± 0.30	3.67 ± 0.49
Neutrophils (%)	51.33 ± 2.11	50.68 ± 2.12
Lymphocytes (%)	44.45 ± 3.55	43.21 ± 3.34
Mean corpuscular volume (%)	64.65 ± 2.23	66.14 ± 2.11
Mean corpuscular hemoglobin (pg/cell)	23.72 ± 0.56	24.65 ± 0.68
Mean corpuscular hemoglobin conc. (%)	32.51 ± 1.36	34.35 ± 1.12

Results represent the mean ± S.D.

**Table 5:** Kidney, liver, and lipid profiles.

Biochemical Analysis	Group A (Control)	Group B (Treated)
ALT (IU/L)	137.14 ± 1.31	145.35 ± 1.46
AST (IU/L)	37.60 ± 3.21	39.27 ± 2.46
Urea (mmol/L)	15.87 ± 0.31	17.40 ± 1.22
Creatinine (mg/dL)	1.21 ± 0.20	1.28 ± 0.31
Uric acid (mg/dL)	4.04 ± 1.24	3.64 ± 1.71
Cholesterol (mg/dL)	64.50 ± 2.32	62.23 ± 2.10
Triglycerides (mg/dL)	61.55 ± 2.45	59.35 ± 3.70

Results represent the mean ± S.D.

**Table 6:** Pharmacokinetic parameters of pure drug and drug-loaded polymeric network.

Formulations	pK Parameters					
	C_max_ (µg/mL)	t_max_ (h)	t_1/2_ (h)	AUC_0-∞_ (µg h/mL)	V_d_	MRT (h)
CAP oral solution	4.44	2	1.27	10.87	1.68	3.02
Drug-loaded hydrogels	3.64	3	13	42.88	4.62	16.09

**Table 7:** Composition of Chitosan/Fenugreek-g-poly (MAA) Hydrogels.

Code	Chitosan (g)	Fenugreek (g)	MAA (g)	MBA (g)	APS (g)
CHF1	1	0.3	15	0.18	0.15
CHF2	2	0.3	15	0.18	0.15
CHF3	3	0.3	15	0.18	0.15
CHF4	1	0.5	15	0.18	0.15
CHF5	1	0.7	15	0.18	0.15
CHF6	1	0.9	15	0.18	0.15
CHF7	1	0.3	20	0.18	0.15
CHF8	1	0.3	25	0.18	0.15
CHF9	1	0.3	30	0.18	0.15
CHF10	1	0.3	15	0.2	0.15
CHF11	1	0.3	15	0.22	0.15
CHF12	1	0.3	15	0.24	0.15

## Data Availability

All data are reported in the manuscript.
